# Clinical, laboratory and genetic characteristics of VEXAS syndrome: a study on behalf of GESMD

**DOI:** 10.3389/fimmu.2026.1743509

**Published:** 2026-04-29

**Authors:** Marta Fonseca-Santos, Borja Puertas, Javier Arzuaga-Méndez, Idoya Ancin-Arteaga, Sandra Castaño-Diez, Carlos De Miguel, Marta Canet-Maldonado, Marc Bosch-Schips, Pablo Palomo-Rumschisky, María Laura Blanco, Daniel Gil-Alós, María Moreno-Igoa, Pedro G. Santa-Catalina, Raúl Azibeiro, Carlos Puerta, Alba Mesa-Tudela, Felix López-Cadenas, Esperanza Tuset, Dolly Viviana Fiallo-Suarez, Fernando Ramos, Maria Teresa Gimenez, Valeria Peri, Karan Mayani, M. Ángeles Montañes-García, Ana Villalba, Jesus María Hernandez Rivas, Marina Díaz-Beyá, María Díez-Campelo

**Affiliations:** 1 Servicio de Hematología, Hospital Universitario de Salamanca (HUS), Salamanca, Spain; 2 Instituto de Investigación Biomédica de Salamanca (IBSAL), Salamanca, Spain; 3 Centro de Investigación del Cáncer de Salamanca (CIC-IBMCC), Madrid, Spain; 4 Universidad de Salamanca (USAL), Salamanca, Spain; 5 Grupo Español de Síndromes Mielodisplásicos (GESMD), Madrid, Spain; 6 Hematology Department. Biocruces Bizkaia Health Research Institute, Hospital Universitario Cruces, Barakaldo, Bizkaia, Spain; 7 Hematology Department. Hospital Clínic de Barcelona, Institut d’Investigacions Biomèdiques August Pi i Sunyer (IDIBAPS), Barcelona, Spain; 8 Hematology Department, Hospital Universitario de Alava - Sede Txagorritxu, Vitoria-Gasteiz, Spain; 9 Hematology Department, Hospital Universitari Mútua Terrassa, Terrassa, Spain; 10 Hematology Department, Vall d’Hebron Hospital Universitari, Experimental Hematology, Vall d’Hebron Institute of Oncology (VHIO), Vall d’Hebron Barcelona Hospital, Barcelona, Spain; 11 Hematology Department, Hospital Ramón y Cajal, Madrid, Spain; 12 Hematology Department, Hospital de la Santa Creu i Sant Pau, Barcelona, Spain; 13 Department of Hematology, Hospital Universitario 12 de Octubre, Instituto de Investigación Sanitaria Hospital 12 de Octubre (imas12), CNIO, CIBERONC, Madrid, Spain; 14 Servicio de Genética, Hospital Universitario de Navarra, Pamplona, Spain; 15 Oncology Department, Hospital Universitario de Salamanca, IBSAL, Salamanca, Spain; 16 Hematology Department. Hospital Universitario Germans Trias y Pujol, Badalona, Spain; 17 Department of Hematology Laboratory; Institut Català Oncologia (ICO), Hospital Doctor Josep Trueta, Girona, Spain; 18 Hematology Department, Hospital Universitario de Gran Canaria Dr. Negrín, Las Palmas de Gran Canaria, Spain; 19 Hematology Department, Hospital de León, León, Spain; 20 Hematology Department, Hospital Verge de la Cinta de Tortosa, Tortosa, Spain; 21 Hematology Department, Complejo Hospitalario Insular, Las Palmas de Gran Canaria, Spain; 22 Hematology Department, Hospital Universitario de la Palma, Santa Cruz de Tenerife, Spain; 23 Hematology Department, Hospital Miguel Servet, Zaragoza, Spain; 24 Hematology Department, Hospital de Sagunto, Valencia, Spain; 25 Centro de Investigación Biomédica en Red en Cáncer (CIBERONC), Madrid, Spain

**Keywords:** autoinflammatory disease, clonal hematopoiesis, myelodysplastic syndromes, UBA1 gene, VEXAS syndrome

## Abstract

VEXAS syndrome (‘Vacuoles’, ‘E1 enzyme’, ‘X-linked’, ‘Autoinflammatory’ and ‘Somatic’) is a rare autoinflammatory disorder caused by somatic mutations in the *UBA1* (ubiquitin-like modifier-activating enzyme 1) gene whose treatment and prognosis remain poorly understood. The GESMD group (Grupo Español de Síndromes Mielodisplásicos) conducted a retrospective multicenter study of 40 male patients (median age 74 years), to analyze clinical and biological features, treatments, responses, prognosis and outcomes. *UBA1* mutations were present in all patients, the c.209T>A mutation—previously reported by García-Escudero et al. as a novel and presumably causative variant in VEXAS syndrome—was associated with older age at diagnosis, a lower frequency of lymph node involvement, and milder anemia. However, these data should be validated in larger cohorts. Overall, 58 treatment lines were administered, including corticosteroids, JAK inhibitors, anti-IL1 and anti-IL6 agents, hypomethylating agents (HMA), and other immunomodulators. Corticosteroid therapy led to rapid early improvement but limited by dependence and tapering difficulties. HMA showed a gradual and sustained increase in global, clinical, and biological responses over time, reaching high proportions of improvement at 12 months despite being used predominantly in more refractory patients (83.3% of patients showed a global response, including 50.0% of complete responses and 33.3% of partial responses). The median time to next treatment or death (TNT-D) was 11 months. Treatment discontinuation occurred in 25 of 58 lines (43.1%), mainly due to lack of efficacy, toxicity, or the need for steroid-sparing. Overall survival data remained immature due to the limited number of events, although gastrointestinal involvement appeared associated with poorer prognosis (*P* = 0.018), while achieving clinical or biological response correlated with better survival (*P* = 0.001 and *P* = 0.003). This study provides a detailed characterization of the Spanish VEXAS cohort, highlighting evolving therapeutic trajectories and the potential role of HMA, and underscores the need for larger prospective studies to define long-term outcomes.

## 1 Introduction

VEXAS (Vacuoles, E1 enzyme, X-linked, Autoinflammatory, Somatic) syndrome is a recently identified autoinflammatory disease caused by somatic mutations in *UBA1*, which encodes the E1 ubiquitin-activating enzyme on the X chromosome. These mutations impair ubiquitination, clonal hematopoiesis, and dysregulated innate immune activation, resulting in systemic inflammation (1, 2). Clinically, VEXAS combines autoinflammatory features—such as fever, neutrophilic dermatosis, chondritis, and arthritis—with hematologic manifestations including cytopenias, thrombosis, myelodysplastic syndromes (MDS) or monoclonal gammopathies, often accompanied by cytoplasmic vacuoles or dysplasia in bone marrow (BM) precursors (3–5).

As *UBA1* is X-linked, initial descriptions included only men with p.Met41 mutations. Subsequent studies have identified additional pathogenic variants, such as p.Ser56Phe and splice-site mutations (c.118-1G>C) (6, 7), and cases have also been reported in women with monosomy X (8, 9). Although *UBA1* variants were initially considered fully penetrant, recent evidence shows asymptomatic carriers and reduced penetrance (10). The mechanisms linking *UBA1* mutations to the inflammatory and hematologic phenotype remain incompletely understood, but specific associations have been described, including the coexistence of polychondritis with MDS and the distinct BM profile of p.Ser56Phe carriers (increased erythropoiesis and reduced granulopoiesis) (6, 11).

The clinical presentation of VEXAS syndrome is highly heterogeneous. Fever is the most common manifestation and usually responds to increased doses of glucocorticoids. Other frequent features include non-vasculitic skin lesions—most often neutrophilic dermatosis—vasculitis of small, medium, or large vessels, arthritis, chondritis, ocular inflammation, and venous thromboembolism, including deep vein thrombosis, pulmonary embolism, and superficial thrombosis (1, 12–14).

The prognosis of VEXAS syndrome remains incompletely defined, with reported mortality rates ranging widely from 15.5% to 40% (1, 5, 15). Factors associated with poorer outcomes include concomitant MDS, transfusion dependence, severe thrombocytopenia, gastrointestinal involvement, pulmonary infiltrates, mediastinal lymphadenopathy, and the *UBA1* p.Met41Val variant (5, 16, 17). The absence of standardized therapeutic strategies, together with treatment-related immunosuppression and the complex clinical profile of affected patients, contributes to suboptimal outcomes in a subset of cases.

The therapeutic management of VEXAS syndrome remains challenging, often requiring the involvement of multiple medical specialties, and current strategies are largely extrapolated from other rheumatologic conditions, with glucocorticoids constituting the usual first-line therapy (18, 19). Disease-modifying antirheumatic drugs and targeted biologic agents have been used with variable clinical benefit (20–22). More recently, JAK inhibitors, particularly Ruxolitinib, have shown efficacy in controlling inflammatory manifestations and cytopenias, both in patients with and without concomitant MDS (23, 24). Azacitidine has also demonstrated benefit in VEXAS associated with MDS, as well as in non-MDS cases, although evidence in the latter remains limited (24–27).In this context, new guidance statements are currently being established by expert groups to support diagnosis and management (18).

Against this background, the Grupo Español de Síndromes Mielodisplásicos (GESMD) designed a retrospective study to describe the clinical and biological characteristics; to analyze the treatment used and its efficacy; and to determine the prognosis and identify prognostic factors of patients with VEXAS syndrome.

## 2 Materials and methods

### 2.1 Patients

A retrospective observational multicenter study was conducted, including 40 patients with genetically confirmed VEXAS syndrome across 17 Spanish Hospitals from September 2022 to October 2023. The follow-up cut-off date was 1st November 2023.

### 2.2 Molecular assessment

*UBA1* mutation detection was performed by Sanger sequencing on BM samples from 39 patients and in peripheral blood in one patient. Next generation sequencing (NGS) was performed on 30 BM samples allowing the identification of mutated genes. The clinical and biological characteristics of the patients at the time of diagnosis of VEXAS syndrome were collected and concomitantly the *UBA1* gene study was requested.

### 2.3 Clinical assessment and study endpoints

Diagnosis of different hematological and rheumatological diseases was made according to World Health Organization (28, 29) and to European Alliance of Associations for Rheumatology and the American College of Rheumatology, respectively. Assessment of treatment response was performed according to the new criteria described by Hadjadj et al. (30) and Al-Hakim et al (21). According to their definitions, complete response (CR) was defined by clinical remission (absence of clinical symptoms related to VEXAS), C reactive protein (C-RP) ≤ 10mg/L (same as ≤ 1mg/dL) and a ≤ 10mg/day of prednisone or equivalent therapy and partial response (PR) was defined by clinical remission and a 50% reduction of C-RP and prednisone or equivalent therapy. In addition, secondary response assessments were performed, as Heiblig et al (23) described, Clinical response (CR) was defined as improvement and/or resolution of clinical symptoms, being partial (CPR) or complete (CCR). Biological response (BR) was defined as a reduction of at least 50% or normalization of C-RP level, being partial (PBR) or complete (CBR) respectively.

Time to next treatment or death (TNT-D) was defined as the time between treatment initiation and the date of next subsequent treatment initiation or death, whichever occurs first. TNT-D survival therefore represents the survival free from these events. two overall survival endpoints were defined: OS-Mutation, calculated from *UBA1* mutation detection to death, and OS-Symptom, calculated from the onset of the first symptom to death.

### 2.4 Statistical analysis

A descriptive analysis of clinical and biological characteristics of patients with VEXAS syndrome was performed. Quantitative variables with normal distribution were expressed as mean and range, whereas non-normally distributed variables were expressed as median and interquartile range (IQR) and qualitative variables as frequency and percentage.

An association was established between the genotype and the clinical and biological features present, if found in more than 10% of the patients included in the study. Chi-square tests identified statistically significant differences between categories of qualitative variables; the associated odds ratio (OR) and 95% CI were estimated by logistic regression.

TNT-D and OS were analyzed using the non-parametric test Kaplan-Meier estimator and groups were compared using the log-rank test, including 95% confidence intervals (CI). Univariable associations were studied including age at diagnosis, p.Met41 mutations, organ involvement, associated diseases, grade ≥2 cytopenia, transfusion dependence, inflammatory biomarkers, therapeutic approaches and response to treatment, multivariable analyses were carried out fitting Cox proportional Hazard models, including the most significant clinical variables. Variables that correlated significantly with different endpoints in the univariate analysis were entered into the multivariate analysis, and only those with *P*<0.05 were retained. SPSS (IBM, SPSS Statistics for Windows, version 21.0. Armonk, NY) and R software were used for all analyses.

## 3 Results

### 3.1 Characteristics of patients with VEXAS syndrome: clinical, associated disorders and biologic features

Forty patients were diagnosed with an autoinflammatory syndrome with confirmed *UBA1* mutation in the period of study. The median age at the time of confirmatory diagnosis was 74 years (range, 47-86 years), and all patients were male. Nine were diagnosed in 2021, 23 in 2022 and eight patients in 2023. The first presenting symptom at the onset of the VEXAS syndrome was cutaneous in 42.5% (n=17) and rheumatologic in 30.0% of patients (n=12). **Supplementary Table 1**.

The most reported symptoms were constitutional syndrome in 90% of patients: asthenia (n=29, 72.5%), fever (n=26, 65.0%) and weight loss (n=13, 32.5%). Skin was the most frequently affected organ in 82.5% of patients (n=33), with neutrophilic dermatitis being the most frequent manifestation (50.0%, n=20). 75% (n=30) percent of patients had rheumatological symptomatology, 57.5% (n=23) had pulmonary involvement, 37.5% (n=15) of patients developed ocular symptomatology and 35% (n=14) had arterial or venous thrombosis. The median number of organs and/or systems affected was 4 (range, 1-7). These patterns, with prominent constitutional, cutaneous and rheumatologic involvement, are in line with previously reported (12). The remaining clinical characteristics of the patients included in the study are detailed in **Table 1**.

**Table 1:** Clinical characteristics.

Clinical characteristic *	All patients (N=40)
Median age, years (range) ≥70 years, n (%)	74 (47-86) 23 (57.5)
Male, n (%)	40 (100)
Median organ/system involvement (range) >4, n (%)	4 (1-8) 11 (27.5)
**General syndrome, n (%)** Asthenia, n (%) Fever, n (%) >10% of weight loss in 6 months, n (%)	36 (90.0) 29 (72.5) 26 (65.0) 13 (32.5)
**Cutaneous, n (%)** Neutrophilic dermatitis, n (%) Vasculitis**, n (%) Erythema nodosum, n (%) Non-specific skin lesions, n (%)	33 (82.5) 19 (47.5) 7 (17.5) 3 (7.5) 4 (10.0)
**Chondroarticular involvement, n (%)** Arthritis and/or arthralgias, n (%) Auricular and or nasal chondritis, n (%)	30 (75.0) 24 (60.0) 19 (47.5)
**Pulmonary, n (%)** No infectious pulmonary infiltrates***, n (%) Pleural effusion, n (%) Diffuse alveolar hemorrhage, n (%)	23 (57.5) 19 (47.5) 5 (12.5) 1 (2.5)
**Ocular, n (%)** Conjunctivitis, n (%) Scleritis, n (%) Uveitis, n (%) Periorbital edema, n (%) Orbital mass, n (%)	15 (37.5) 6 (15.0) 4 (10.0) 3 (7.5) 1 (2.5) 1 (2.5)°
**Unprovoked thrombosis, n (%)** Venous, n (%) Arterial, n (%)	14 (35.0) 13 (32.5) 5 (12.5)
**Adenopathy and visceromegaly, n (%)** Lymph nodes, n (%) Splenomegaly, n (%)	13 (32.5) 9 (22.5) 7 (17.5)
**Cardiac, n (%)** Acute myocardial infarction, n (%) Pericarditis, n (%) Myocarditis, n (%)	4 (10.0) 2 (5.0) 1 (2.5) 1 (2.5)
**Digestive, n (%)** Gastrointestinal bleeding, n (%) Abdominal pain, n (%) Intestinal perforation, n (%)	4 (10.0) 2 (5.0) 1 (2.5) 1 (2.5)
**Arterial, n (%)** Aortitis, n (%) Aneurisms, n (%)	3 (7.5) 3 (7.5) 2 (5.0)
**Peripheral nervous system involvement, n (%)** Sensory neuropathy, n (%) Multiple mononeuropathy, n (%)	3 (7.5) 2 (5.0) 1 (2.5)
**Renal, n (%)** Nephritis, n (%)	2 (5.0) 2 (5.0)
**Testicular, n (%)** Orchiepididymitis, n (%)	2 (5.0) 2 (5.0)

*It should be noted that there were patients who may have more than one symptomatology in the same organ/system.

**Vasculitis included livedo reticularis (n=7), leukocytoclastic vasculitis (n=4) and necrotizing vasculitis (n=1).

***Noninfectious pulmonary infiltrates includes non-specific infiltrates (n=12), organizing pneumonia (n=3) and interstitial pneumonia (n=2).

Regarding the biological and hematological characteristics, it is noteworthy that the median hemoglobin was 9.81 g/dL (IQR, 8.35 – 10.87 g/dL), predominantly macrocytic, 62.5% (n=25) of patients had grade ≥2 anemia and 27.5% of patients (n=11) had transfusion dependency. However, other grade ≥2 cytopenia are described in **Table 2**, without being as relevant as in the previous scenario. Patients showed elevated levels of inflammatory biomarkers such as erythrocyte sedimentation rate (ESR), ferritin and C-RP. Regarding the characteristics observed in the BM morphological study, about 60% (n=20) were hypercellular, 66% (n=26) had dysplastic abnormalities, and most of smears showed intracytoplasmic vacuoles in the immature erythroid and/or myeloid series (n=36, 94.7%) **Table 2**.

**Table 2:** Biological characteristics’.

Biological characteristics All patients (N=40)	
Blood cell counts	
Hemoglobin (g/dL), mean (range) ≥Grade 2 anemia	9.80 (6.90 – 14.20) 25 (62.5)
Mean corpuscular volume (fL), mean (range)	104.85 (88.4 – 127.0)
Neutrophils (x10^3^/uL), median (IQR) > Grade 2 neutropenia, n (%)	3.55 (2.30 – 4.68) 2 (5.0)
Lymphocytes (x10^3^/uL), median (IQR) > Grade 2 lymphopenia, n (%)	1.12 (0.77-1.70) 11 (27.5)
Platelets (x10^3^/uL), median (IQR) > Grade 2 thrombocytopenia, n (%)	135.50 (88.50 – 202.00) 7 (17.5)
Inflammatory biomarkers	
Erythrocyte sedimentation rate (mm/h), median (IQR)	93.00 (47.00-129.00)
C-reactive protein (mg/dL), median (IQR)	8.28 (4.00-13.41)
Ferritin (ng/mL), median (IQR)	625.00 (334.00 – 1695.00)
Lactate dehydrogenase (UI/L), median (IQR)	203.00 (160.75 – 282.25)
Bone marrow*	
**Cellularity** Hypercellular, n (%) Normocellular, n (%) Hypocellular, n (%)	Reported in 33 of 39 20/33 (60.6) 12/33 (36.4) 1/33 (2.5)
**Dysplastic** Myeloid, n (%) Erythroid, n (%) Megakaryocytic, n (%) Single lineage, n (%) Multiple lineages, n (%) No, n (%)	Reported in 39 of 39 18/39 (46.2) 18/39 (46.2) 11/39 (28.2) 11/39 (27.5) 15/39 (38.5) 13/39 (33.3)
**Vacuoles** Myeloid, n (%) Erythroid, n (%) No, n (%)	Reported in 38 of 39 36/38 (94.7) 13/38 (34.2) 2/38 (5.3)
Blasts in %, median (IQR)	2.24 (0-2)
Type of *UBA1* mutation, n (%)	
c.122T>C (p.Met41Thr) c.121A>C (pMet41Leu) c.121A>G (pMet41Val) Others	16 (40.0) 12 (30.0) 6 (15.0) 6 (15.0)

*Bone marrow aspiration/biopsy was not performed in one patient.

All patients with VEXAS syndrome also met diagnostic criteria for rheumatological or hematological diseases, occurring in 36 and 24 patients, respectively, and 20 patients had both manifestations. The median number of associated rheumatological diseases was 2 (range, 1-4), being Sweet’s syndrome (n=20) and relapsing polychondritis (n=16) the most reported entities. Regarding associated hematological diseases, the median was 1 (range, 1-2), 18 patients (45.0%) had a previous diagnosis of MDS: 15 were MDS with low blasts (12 with multilineage dysplasia (MDS-LB-MLD) and 3 with single lineage dysplasia (MDS-LB-SLD)) and 3 patients were MDS with increased blasts type 1 (MDS-IB-1), in line with previously reported data (12) (**Table 3**).

**Table 3:** Rheumatologic and Hematologic diseases.

Rheumatologic diseases (N = 36)*	
Sweet Syndrome, n (%)	20 (55.6)
Relapsing polychondritis, n (%)	16 (44.4)
Seronegative arthritis, n (%)	9 (25.0)
Polyarteritis nodosa, n (%)	8 (22.2)
Non-Specified Rheumatologic Syndrome, n (%)	4 (11.1)
Polymyalgia rheumatica, n (%)	3 (8.3)
Systemic lupus erythematosus, n (%)	1 (2.8)
Giant cell arteritis, n (%)	1 (2.8)
Hematologic disease (N = 24)	
Myelodysplastic syndrome with low blast and multilineage dysplasia (MDS-LB-MLD)	12 (50.0)
Myelodysplastic syndrome with low blast and single lineage dysplasia (MDS-LB-SLD)	3 (12.5)
Myelodysplastic syndrome with increased blasts type 1 (MDS-IB1)**	3 (12.5)
Monoclonal gammopathy of undetermined significance (MGUS)	2 (12.5)
Clonal cytopenia of undetermined significance (CCUS)	1 (4.2)
Monoclonal B-cell lymphocytosis (MBL)	1 (4.2)
Hodgkin lymphoma (HL)	1 (4.2)
Acute Myeloid Leukemia (AML)	1 (4.2)

*Patients could have more than one rheumatological disease.

**One patient had MGUS and MDS-IB1.

### 3.2 Mutations shown in VEXAS syndrome: frequency, co-mutations and genotype-phenotype correlations

Thirty-five patients had p.Met41 mutations: p.Met41Thr (Thr) in 16 (40.0%), p.Met41Leu (Leu) in 12 (30.0%), p.Met41Val (Val) in six (15.0%) and an unknown p.Met41 was observed in one patient (2.5%). The remaining mutations were splicing mutations: c.118-1G>3 (n=3, 7.5%) and c.346-2A>G (n=1, 2.5%) (**Table 2**). In a patient with a compatible clinical presentation, the c.209T>A mutation (n=1, 2.5%) was identified and described as a variant of uncertain significance, which may potentially affect protein function and had been previously reported by García-Escudero et al. (31).

NGS was performed in 30 patients. The median number of co-mutations in our series was 0 (range, 0-3) and the isolated *UBA1* mutation was the most frequent genomic landscape. Within the ten patients with co-mutations, seven were patients with a prior diagnosis of MDS, and *TET2* (n=4) and *DNMT3A* (n=3) were the most detected mutations. **Supplementary Table 2**.

Exploratory comparisons between *UBA1* p.Met41 variants and clinical characteristics were performed. Patients with the Thr mutation showed higher frequencies of relapsing polychondritis (75.0% vs. 16.7%, OR 15.0 [95% CI, 3.15-71.36]; *P*<0.001), ocular involvement (56.3% vs. 25.0%, OR 3.85 [95% CI, 1.10-14.96]; *P* = 0.046), and a trend toward more pulmonary involvement (75.0% vs. 45.8%, OR 3.54 [95% CI, 0.88-14.19]; *P* = 0.068). The Leu mutation was more frequent in older patients (>70 years: 83.3% vs. 46.4%, OR 5.8 [95% CI, 1.10-31.30]; *P* = 0.042). Lymphadenopathy appeared less common in patients with the Leu variant (0.0% vs. 32.1%, OR not estimated, *P* = 0.026), as well as grade ≥2 anemia (25.0% vs. 78.6%, OR 0.09 [95% CI, 0.01-0.44]; *P* = 0.003). No specific phenotype was observed in patients with the Val mutation. No statistically significant differences were detected between mutation type and MDS, Sweet syndrome, transfusion dependence, other clinical features, or inflammatory biomarkers. Given the small number of patients within each mutation subgroup, all genotype–phenotype comparisons should be considered exploratory, interpreted with caution, and validated in larger, independent cohorts.

Although the c.209T>A mutation is classified as a variant of uncertain significance and further studies are ongoing, the clinical suspicion of this patient was triggered by the debut of polyarteritis nodosa in a patient with MDS-LB-MLD. The patient also had fever, weight loss, asthenia, bilateral pleural effusion, multiple mononeuritis, and non-transfusion-dependent grade 2 anemia, along with morphological features in the BM exam compatible with VEXAS syndrome.

### 3.3 Treatment of VEXAS syndrome

Twenty-three patients (57.5% of the cohort) received some form of immunosuppressive therapy before the genetic diagnosis was made, with a median of 2 drugs (range, 0-6). The most frequently used agents were glucocorticoids and methotrexate (91.3% (n=21) and 56.5% (n=13), respectively), and to a lower measure azathioprine, anti-IL6 or anti-IL1 among others. However, 69.6% (n=16) of previously treated patients changed the therapy after the *UBA1* mutation was identified.

**Figure 1:**
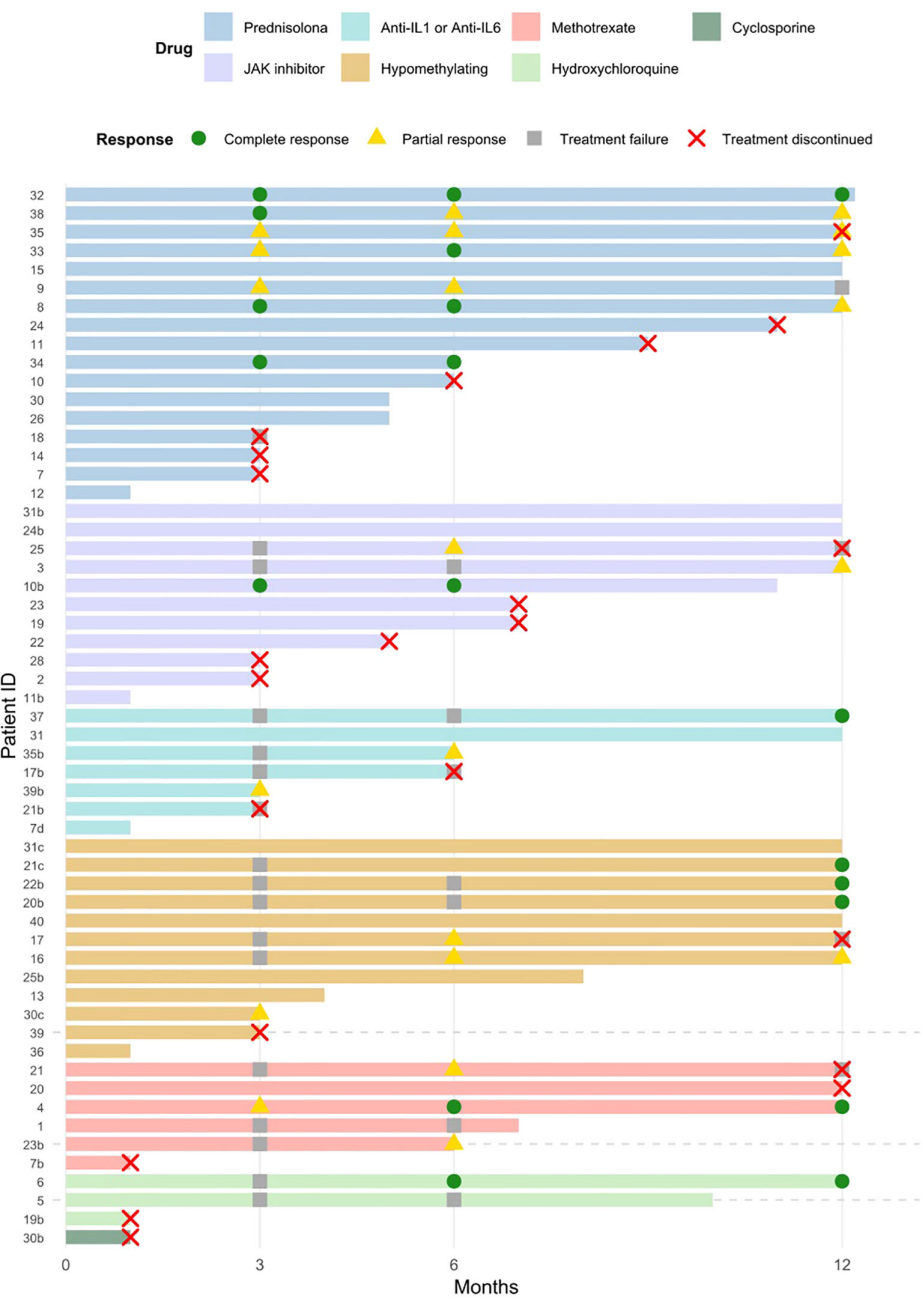
Individual patient responses to different treatments. Swimmer plot showing treatment duration and response status at 3, 6 and 12 months. Each bar represents a patient, and symbols denote response at each time point.

After genetic diagnosis, 39 patients were treated (one patient did not start treatment at the time of analysis) and the median number of lines of therapy was 1 (range, 1-3), with a median follow up after diagnosis of 12.5 months (IQR, 4-17 months). One patient directly underwent allogeneic stem cell transplantation due to underlying MDS with increase of blast type 1.The rest of treatments used after genetic diagnosis were heterogeneous, which, for simplicity and affinity were stratified into four groups (**Table 4**; **Figure 1**). Across the 38 patients included in the study, a total of 58 treatment lines were administered. These treatment lines were subsequently stratified into four therapeutic categories (**Table 4**). Steroid-monotherapy (n=17, 29.3%), JAK inhibitor–based therapy (n=12, 20.7%) including 1 patient with upadacitinib, 1 patient with baricitinib and 9 patients with ruxolitinib, anti-IL (n=7, 12.1%) including 2 patients with Tocilizumab (anti-IL6), 1 patient with canakinumab and 2 patients with anakinra (both IL-1 inhibitors), HMA therapy (n=12, 20.7%) 1 patient with decitabine and the rest treated with azacitidine, and other immunomodulatory approaches (n=10, 17.2%), including 6 patients treated with methotrexate, 3 with hydroxychloroquine and 1 with cyclosporine. A detailed description of all treatment lines, including line of therapy and adjusted percentages, and steroids initial dose is provided in **Supplementary Table 3**.

**Table 4:** Treatment and response in VEXAS syndrome cohort.

Characteristics All patients N = 38 Overall treatment lines= 58	Steroid-monotherapy N = 17 (31.0%)	Jak inhibitor-based therapy N=11 (19.0%)	Anti-IL1 and 6 based therapy N= 7 (12.1%)	Hypomethylating based therapy N=12 (20.7%)	Others N=10 (17.2%)
At therapy initiation					
Previous rheumatological diagnosis Sweet Syndrome, n (%) Relapsing polychondritis, n (%) Seronegative arthritis, n (%) Non-specified Rheumatologic Syndrome, n (%) Polyartheritis nodosa, n (%) Systemic Lypus erythematosus, n (%) Giant cell arteritis, n (%)	11/17 (64.7) 7/17 (41.2) 3/17 (17.6) 4/17 (23.5) 3/17 (17.6) 1/17 (5.9) 0/17 (0)	3/11 (27.3) 8/11 (72.7) 3/11 (27.3) 3/11 (27.3) 0/11 (0) 0/11 (0) 0/11 (0)	1/7 (14.3) 5/7 (71.4) 1/7 (14.3) 1/7 (14.3) 1/7 (14.3) 0/7 (0) 0/7 (0)	3/12 (25.0) 8/12 (66.7) 4/12 (33.3) 0/12 (0) 2/12 (16.7) 0/12 (0) 1/12 1 (8.3)	2/10 (20.0) 5/10 (50.0) 0/10 (0) 0/10 (0) 3/10 (30.0) 0/10 (0) 0/10 (0)
Previous haematological diagnosis MDS-LB-SLD MDS-LB-MLD MDS-IB1 MGUS CCUS MBL HL AML	2/17 (11.8) 4/17 (23.5) 1/17 (5.9) 2/17(11.8) 0/17 (0) 0/17 (0) 0/17 (0) 0/17 (0)	3/11 (27.3) 3/11 (27.3) 0/11 (0) 1/11 (9.1) 0/11 (0) 0/11 (0) 0/11 (0) 0/11 (0)	0/7 (0) 2/7 (28.6) 0/7 (0) 0/7 (0) 0/7 (0) 0/7 (0) 1/7 (14.3) 0/7 (0)	1/12 (8.3) 5/12 (41.7) 0/12 (0) 0/12 (0) 1/12 (8.3) 0/12 (0) 0/12 (0) 1/12 (8.3)	0/10 (0) 3/10 (30.0) 0/10 (0) 0/10 (0) 0/10 (0) 1/10 (10.0) 0/10 (0) 0/10 (0)
C-RP mg/dL, median (IQR)	4.60 (2.12-9.82)	10.38 (6.48-21.92)	6.93 (3.67-13.22)	7.87 (4.0-16.32)	10.01 (7.2-16.4)
Haemoglobin g/dL, mean (range)	10.07 (8.00-13.60)	9.50 (6.9-11.4)	10.41 (6.9-14.2)	9.49 (6.9-13.60)	9.6 (7.5-13.6)
Transfusion dependency, n (%)	2/17 (11.1)	4/11 (36.4)	3/6 (50.0)	6/11 (50.0)	1/10 (10)
Concomitant glucocorticoids at therapy initiation, n (%)	NA	7/7 (100)	5/5 (100)	9/11 (81.8)	8/8 (100)
Prednisolone (or equivalent), dose (mg/day), median (IQR)	30 (10-80)	10 (26-52.5)	10 (5-35)	20 (5-60)	30 (10-60)
Previous lines of treatment, n (range)	1 (1-3)	3 (2-4)	3 (2-4)	3 (1-8)	3 (1-4)
Therapeutic line First Second Third Fourth	NA NA NA NA	7/11 (63.6) 4/11 (36.4) 0/11 (0) 0/12 (0)	2/6 (33.3) 4/6 (66.7) 0/6 0/6	4/12 (33.3) 3/12 (25.0) 5/12 (41.7) 0/12 (0)	6/10 (60.0) 4/10 (40.0) 0/10 (0) 0/10 (0)
Month 3					
Clinical response, n (%) Complete Partial No response	5/11 (45.5) 2/11 (18.2) 4/11 (36.3)	2/8 (25.0) 3/8(37.5) 3/8 (37.5)	1/5 (20.0) 4/5 (80.0) 0/5 (0)	1/9 (11.1) 4/9 (44.4) 4/9 (44.4)	2/7 (28.6) 5/7 (71.4) 0/7 (0)
Biological response Complete Partial No response	5/11 (45.5) 1/11 (9.1) 5/11 (45.5)	0/6 (0) 1/6 (16.7) 5/6 (83.3)	0/5 (0) 5/5 (100) 0/5 (0)	2/7 (25.0) 1/7 (12.5) 5/7 (62.5)	1/6 (16.7) 2/6 (33.3) 3/6 (50.0)
Prednisolone daily dose ≤ 10mg/D >10mg/D	6/9 (66.7) 3/9 (33.3)	1/5 (20.0) 4/5 (80.0)	3/5 (60.0) 2/5 (40.0)	3/8 (37.5) 4/8 (62.5)	3/6 (50.0) 3/6 (50.0)
C-RP, mg/dL ≤ 1 >1	5/9 (55.6) 4/9 (44.4)	0/5 (0) 5/5 (100)	1/5 (20.0) 4/5 (80.0)	2/7 (28.6) 5/7 (71.4)	1/6 (16.7) 5/6 (83.3)
Haemoglobin g/dL, mean (range)	12.22 (9.90-14.60)	7.5 (6.5-9.8)	12.6 (9.6-17.0)	10.1 (7.6-15.8)	8.8 (7.0-11.9)
Transfusion dependency, n (%)	0 (0)	0 (0)	1/5 (20.0)	5/8 (41.7)	2/7 (28.6)
Global response Complete Partial No response	4/9 (44.4) 3/9 (33.3) 2/9 (22.2)	1/7 (14.3) 1/7 (14.3) 5/7 (71.4)	0/5 (0) 1/5 (20.0) 4/5 (80.0)	0/8 (0) 3/8 (37.5) 5/8 (62.5)	0/6 (0) 1/6 (16.7) 5/6 (83.3)
Month 6					
Clinical response, n (%) Complete Partial No response	7/10 (70.0) 3/10 (30.0) 0/10 (0)	2/4 (50.0) 2/4 (50.0) 0 (0)	2/3 (66.7) 1/3 (33.3) 0/3 (0)	4/6 (57.1) 3/6 (42.9) 0/6 (0)	3/6 (50.0) 3/6 (50.0) 0/6 (0)
Biological response Complete Partial No response	4/10 (4.0) 5/10 (50.0) 1/10 (10.0)	0/4 (0) 1/4 (25.0) 3/4 (75.0)	0/3 (0) 2/3 (66.7) 1/3 (33.3)	1/7 (14.3) 1/7 (14.3) 5/7 (71.4)	2/6 (33.3) 3/6 (50.0) 1/6 (16.7)
Prednisolone daily dose ≤ 10mg/D >10mg/D	7/7 (100) 0/7 (0)	1/2 (50.0) 1/2 (50.0)	2/3 (33.3) 1/3 (66.7)	4/6 (66.7) 2/6 (33.3)	4/6 (66.7) 2/6 (33.3)
C-RP, mg/dL ≤ 1 >1	4/7 (57.1) 3/7 (42.9)	0/2 (0) 2/2 (100.0)	0/3 (0) 3/3 (100)	0/6 6/6 (100)	2/6 (33.3) 4/6 (66.7)
Haemoglobin g/dL, mean (range)	11.70 (10.3-15.2)	8.0 (7.3-8.7)	12.2 (9.3-16.8)	12.1 (9.70-14.30)	11.96 (9.8-13.9)
Transfusion dependency, n (%)	0 (0)	0 (0)	1/3 (33.3)	0 (0)	1/6 (16.7)
Global response Complete Partial No response	4/7 (57.1) 3/7 (42.9) 0/7 (0)	1/4 (25.0) 2/4 (50.0) 1/4 (25.0)	0/3 (0) 1/3 (33.3) 2/3 (66.7)	0/6 (0) 3/6 (50.0) 3/6 (50.0)	2/6 (33.3) 2/6 (33.3) 2/6 (33.3)
Month 12					
Clinical response, n (%) Complete Partial No response	6/7 (85.7) 1/7 (14.3) 0/7 (0)	0/3 (0) 3/3 (100.0) 0/3 (0)	1/1 (100) 0/1 (0) 0/1 (0)	3/6 (50.0) 1/6 (16.7) 2/6 (33.3)	2/5 (40.0) 2/5 (40.0) 1/5 (20.0)
Biological response Complete Partial No response	2/7 (28.6) 4/7 (57.1) 1/7 (14.3)	0/3 (0) 3/3 (100) 0/3 (0)	1/1 (100) 0/1 (0) 0/1 (0)	3/6 (50.0) 1/6 (16.7) 2/6 (33.3)	2/5 (40.0) 1/5 (20.0) 2/5 (40.0)
Prednisolone daily dose ≤ 10mg/D >10mg/D	5/5 (100) 0/5 (0)	1/2 (50.0) 1/2 (50.0)	1/1 (100) 0/1 (0)	6/6 (100) 0/6 (0)	2/4 (50.0) 2/4 (50.0)
C-RP, mg/dL ≤ 1 >1	1/5 (20.0) 4/5 (80.0)	0/2 (0) 2/2 (100.0)	1/1 (100) 0/1 (0)	3/6 (50.0) 3/6 (50.0)	2/4 (50.0) 2/4 (50.0)
Haemoglobin g/dL, mean (range)	12.10 (10.6-14.65)	9.7 (7.8-11.6)	17.5 (NA)	12.1 (7.60-15.4)	12.27 (11.8-13.3)
Transfusion dependency, n (%)	0 (0)	1 (50.0)	0 (0)	2/6 (33.3)	0 (0)
Global response Complete Partial No response	1/5 (20.0) 3/5 (60.0) 1/5 (20.0)	0/2 (0) 1/2 (50.0) 1/2 (50.0)	1/1 (100) 0/1 (0) 0/1 (0)	3/6 (50.0) 2/6 (33.3) 1/6 (16.7)	2/4 (50.0) 0/4 (0) 2/4 (50.0)

One patient underwent direct allogeneic stem cell transplantation and one patient did not start treatment at the time of analysis.

All patients in the steroid-monotherapy group (n=17) received this as first-line therapy. In contrast, JAK inhibitor–based therapy was used in 11 patients (19.0%), predominantly as first-line therapy (n=7, 63.6%) and less frequently as second-line therapy (n=4, 36.4%). Anti-IL1/IL6 therapy was used in 7 patients (12.1%), and it was mainly administered as second-line therapy (n=4, 66.7%). HMA therapy was used in 12 patients (20.7%), and was most often employed in later therapeutic lines, including second-line (n=3, 25.0%) and especially third-line therapy (n=5, 41.7%), with only a minority receiving it as first-line treatment (n=4, 33.3%). Other immunomodulatory approaches were used in 10 patients (17.2%), mostly as first-line therapy (6/10, 60.0%) and less frequently as second-line therapy (4/10, 40.0%) (**Table 4**).

Importantly, patients receiving steroid-monotherapy were largely steroid-naïve and had received fewer prior treatment lines (median of 1 prior line [range, 1–3]), whereas those treated with JAK inhibitors, anti-IL1/IL6 agents, HMA therapy or other immunomodulators had typically failed previous corticosteroid courses—with the exception of only two patients in the HMA group who had not received steroids before—thus representing a more treatment-refractory (median treatment lines median of 3 prior lines [ranges 2–4, 2–4, 1–8 and 1–4, respectively]) population and introducing a clear selection bias that limits direct comparisons between groups and subsequent outcomes are presented descriptively.

At 3 months, among the 35 patients with available data, regarding the new response criteria, a CR was achieved in 14.3% of patients (n=5), while 25.7% (n=9) showed a partial response. The majority, 60.0% (n=21), had no clinical response at this early assessment point. In the first-line steroid-monotherapy group, 77.7% (n=7/9) of patients met any response (complete 44.4% [n=4], partial 33.3% [n=3]), reflecting their rapid effect and the fact that all patients in this group received corticosteroids as the first-line treatment. In contrast, early responses were lower in the remaining groups, where most patients had received previous treatments. Any response rates were 28.6% (n=2) for JAK inhibitors, 20.0% (n=1) for anti-IL1/IL6 agents, 37.5% (n=3) for HMA therapy, and 16.7% (n=1) for other immunomodulators, with high proportions of non-responders across all these groups. Complete and partial responses tended to appear earlier in the clinical and biological assessments than in the global response, which is expected given that the global criteria integrate several domains and therefore require a more comprehensive improvement (**Table 4**). Overall, before the initiation of any treatment, transfusion dependency was present in 16 out of 38 patients (42.1%), however, by month 3, this proportion had decreased to 8 out of 38 patients (21.1%), indicating a general reduction in transfusion requirements during early follow-up.

According to the composite global response criteria at month 6, 26 patients were evaluable, of whom 7 (26.9%) achieved a complete response and 11 (42.3%) a partial response, while 8 patients (30.8%) were classified as non-responders. Patients treated with steroid-monotherapy regimens maintained any degree of response according to the composite criteria, with 7 of 7 evaluable patients (100%) showing either a complete or partial response at month 6. In contrast, the remaining therapeutic groups showed a more gradual pattern of improvement, suggesting that other immunosuppressive or disease-modifying treatments may require a longer time to achieve composite responses. Specifically, responses increased from 28.6% to 75.0% in the JAK inhibitor group, from 20.0% to 33.3% with anti-IL1/IL6 agents, from 37.5% to 50.0% with HMA therapy, and from 50.0% 66.7% among the remaining immunosuppressive treatments. Regarding clinical and biological responses, the same trend is observed at month 6, with higher rates than in the composite assessment. In patients receiving non-steroid based therapy, more than 50% achieved a reduction of prednisolone to ≤10 mg/day across all treatment groups, despite being a more heavily pre-treated population. Overall, transfusion dependence improved over time, with a progressive reduction from baseline to month 6 across treatment groups (**Table 4**).

At month 12, global response data were available for 18 patients. A complete response was documented in 22.2% (n=4), while 33.3% (n=6) achieved a partial response; the remaining 44.4% (n=8) were classified as non-responders according to the composite criteria. Steroid-monotherapy regimens showed a slight decline in response rates 80.0% (n=4), suggesting that their initial benefit may attenuate over longer follow-up. In contrast, HMA therapy demonstrated a clear and sustained improvement over time, reaching 83.3% overall responses at month 12 (complete 50.0% [n=3], partial 33.3% [n=2]). The remaining therapeutic groups maintained good response rates, although, data interpretation is limited by the small number of evaluable patients in each category and time point. Clinical and biological responses remained favorable across treatment groups. Clinically, steroid-monotherapy regimens achieved responses in 85.7% of evaluable patients (n=6), anti-IL1/IL6 agents in 100% (n=3), and HMA therapy in 66.7% (n=4). Biological responses followed a similar pattern, with 85.7% (n=6) responding in the steroid-monotherapy group, 100% (n=3) with anti-IL1/IL6 agents, and 66.7% (n=4) with HMA therapy. Steroid-sparing was maintained across treatment groups, with many patients reaching prednisolone doses ≤10 mg/day, though these findings should be interpreted descriptively due to limited sample size. At month 12, transfusion dependency persisted in 3 of the 18 evaluable patients (16.7%), reflecting a continued overall improvement compared with baseline. Overall, clinical and biological responses remained stable in the steroid-monotherapy group, HMA agents continued to show the most pronounced long-term benefit, and the remaining therapeutic groups also maintained meaningful clinical and biological improvement; however, the small number of evaluable patients at this timepoint warrants cautious interpretation.

HMA agents were generally used in later treatment lines and in more treatment-refractory patients, yet they showed a pattern of gradual and sustained improvement, achieving a high proportion of overall responses by month 12. These findings require validation in larger independent cohorts.

Overall, 25 of the 58 treatment lines (43.1%) were discontinued. In the steroid-monotherapy group, discontinuation was mainly driven by corticosteroid-dependence, whereas HMA—despite their known toxicity profile—showed the lowest discontinuation rate (18.2%), highlighting their overall tolerability in this cohort. The median TNT-D in the entire cohort was 4 months (IQR, 1.5-7.5). The median TNT-D differed between therapies, for steroid-monotherapy based therapy was longer (9 months [IQR, 2.5-15.5]), similarly was for HMA based therapy (8 months [IQR, 2-13]), while for JAK based therapy was 7 months [IQR, 3-9.5]), Anti-IL1 and IL6 was 4.5 months [IQR, 1.5-13.5]), and other therapies 5.5 months [IQR, 1-12]) (**Table 5**).

**Table 5:** Treatment discontinuation analysis.

Characteristics All patients N=38 Overall treatment lines= 58	Steroid-monotherapy N=17 (31.0%)	JAK inhibitor-based therapy N=11 (19.0%)	Anti-IL1 and 6 based therapy N= 7 (12.1%)	Hypomethylating based therapy N=12 (20.7%)	Others N=10 (17.2%)
Treatment discontinuation, n (%)	8/17 (47.1)	7/11 (63.3)	3/7 (42.9)	2/11 (18.2)	5/10 (50)
Reason for treatment discontinuation, n (%) Failure to treatment or insufficient control Toxicity Steroid sparing	4 (23.5) 0 (0) 4 (23.5)	7 (63.3) 0 (0) 0 (0)	1 (14.3) 2 (28.6) 0 (0)	1 (8.3) 1 (8.3) 0 (0)	4 (40.0) 1 (10.0) 0 (0)
TNT-D, months (IQR)	9 (2.5-15.5)	7 (3-9.5)	4.5 (1.5-13.5)	8 (2-13)	5.5 (1-12)

### 3.4 Prognosis of VEXAS syndrome

The median time from symptom onset to the genetic diagnosis was 28.5 months (IQR, 11-53 months). A trend towards shorter latency to diagnosis was detected over time: 53 months in patients diagnosed during 2021, and 20 months those diagnosed between 2022 and 2023 (*P* = 0.246).

With a median follow-up of living patients since genetic diagnosis of 12.6 months (IQR, 7.1 – 17.63) the median overall survival (OS) was not reached, the OS at 1 and 2 years was 86.6% (*P* = 95%, 95% CI 85.5-87.6) and 80.4% (*P* = 95%, 95% CI, 78.8-81.9 (**Supplementary Figure 1**). The median follow up of living patients since the first symptom is 53 months (IQR, 17.7 – 81.50).

Six patients died (12%), four (10%) due to infections (three due to sepsis shock and one due to disseminated varicella zoster virus infection), one due to acute myeloid leukemia in progressive disease (2.5%), and the cause of the remaining one was not reported (2.5%).

A preliminary risk-factor analysis for OS was performed (**Supplementary Table 4**). Given the limited number of events, these findings should be interpreted with caution. Gastrointestinal involvement was identified as a risk factor (HR = 8.753, 95% CI 1.444 – 53.044, *P* = 0.018), others such as the presence of MDS, transfusion dependence, severe thrombocytopenia, different *UBA1* mutations or even the pMet41Val variant were not significant in the analysis. Only response to treatment after first line of treatment after diagnosis, both clinical and biological, was identified as a protective factor, as no events occurred in those patients who achieved any response (CR: HR not estimable, *P* < 0.001; and BR: HR not estimable, *P* < 0.001). A similar pattern was observed when OS was analyzed from symptom onset (data not shown). A trend towards lower OS was found in patients with higher levels of inflammatory biomarkers (ESR, C-RP and ferritin) and patients with more severe cytopenia (anemia ≥ 2 and neutropenia ≥ 2). These findings require longer-term follow-up and validation in future studies.

**Figure 2:**
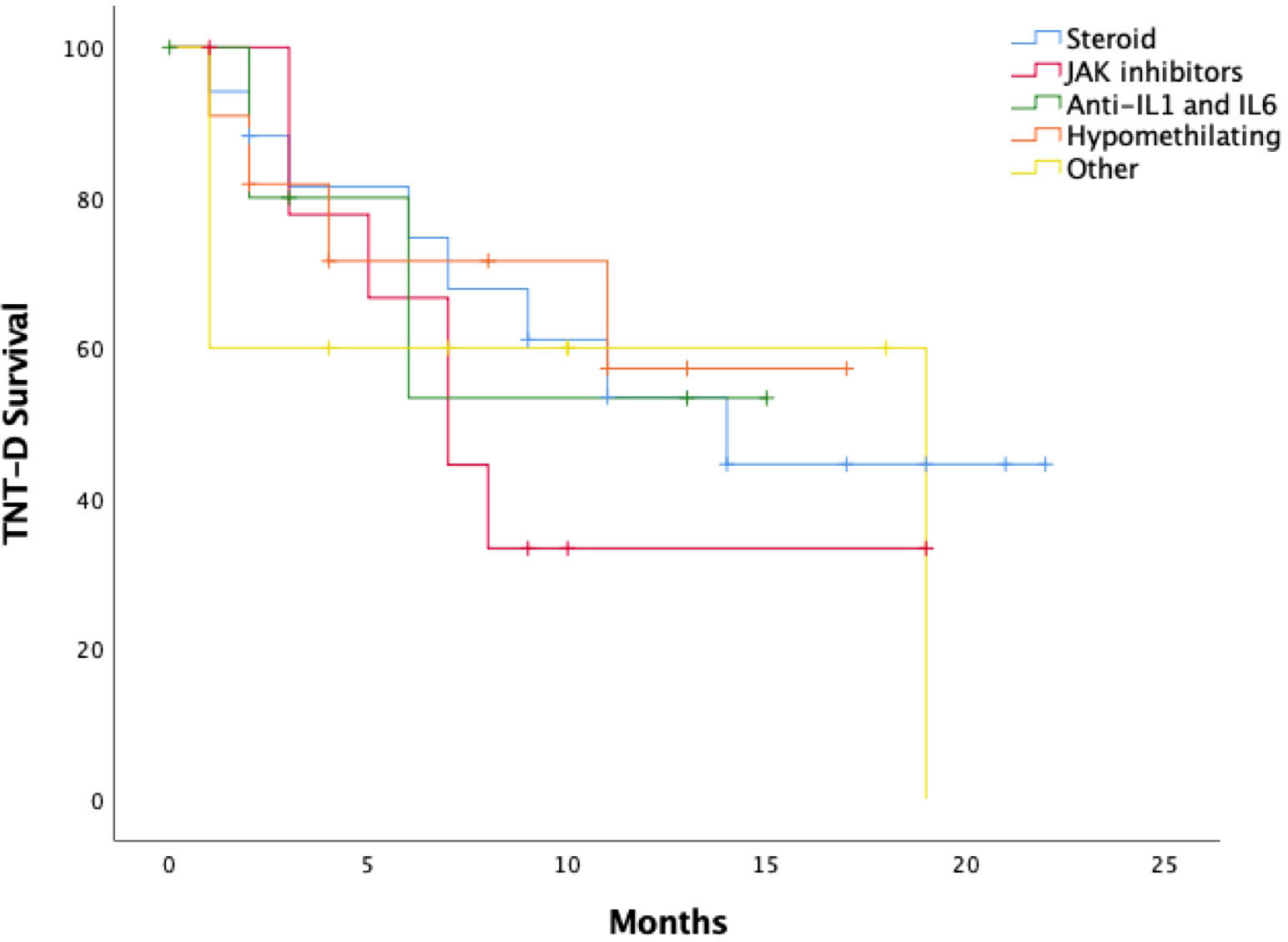
TNT-D Survival including different treatment regimens.

The median TNT-D survival was 11 months. The TNT-D survival at 1 year was 50.0% (*P* = 95%, 95% CI, 34.9-65.1). When comparing between different lines of treatment, no statistical difference was found (*P* = 0.870), being at 1 year of 53.5% (95% CI, 27.8%–79.2%) in the steroid-monotherapy group, 57.3% (95% CI, 24.0%–90.6%) for HMA, 53.3% (95% CI, 4.7%–100%) in the anti-IL1 and IL6 group, 60.0% (95% CI, 29.6%–90.4%) with other treatments and a trend to lower TNT-D survival in the JAK inhibitor group 33.3% (95% CI, 2.5%–64.1%) (**Figure 2**). A risk-factor analysis for TNT-D survival was performed, but no clinical, analytical or genetic variable showed a statistically significant association with the risk of treatment discontinuation or transition to a new line (**Supplementary Table 5**).

## 4 Discussion

This study continues to expand the current knowledge of the heterogeneous clinical and biological characteristics of this entity, as well as the therapeutic possibilities and prognosis of this condition, within a homogeneous multicenter series in Spain.

The clinical features described in our series remain consistent with the initial report of VEXAS syndrome and subsequent studies (1, 12–14, 21, 22, 30). In our series, general symptoms, mainly fever, were found to be predominant (90% and 65%, respectively). In addition, 82.5% and 75.0% of patients had cutaneous and chondroarticular involvement respectively, being the most frequently affected organs, followed by pulmonary (57.7%) and ocular (37.5%) involvement. Cardiac and renal involvement were infrequent in our cohort (10.% and 5.0%), and we did not observe any cases of central nervous system involvement. This overall distribution mirrors the pattern described by Al Hakim et al (12)., where skin disease is the leading manifestation (81.8%) and constitutional, respiratory and ocular symptoms are also common (69.4%, 61.3% and 44.3%), while renal and cardiac involvement are uncommon (7% and 7.6%, respectively). In terms of biological features in the series, the presence of macrocytic anemia and increased inflammatory biomarkers, already widely described in previous studies (5, 21, 22, 30, 32). In addition, BM studies showed a tendency towards hypercellularity and intracytoplasmic vacuoles in the immature erythroid and myeloid series were present in almost all patients, as previously described (1, 3, 33). However, it should be mentioned that two patients in our series did not present this cytological feature, being a typical but not pathognomonic feature (33). In this line, Koster et al. have proposed that the *UBA1* mutation should be considered in men over 40 years or women with monosomy X, with compatible clinical findings and who have an elevated C-RP and require >10 mg/day of prednisone for symptom control, even if there were no vacuoles in the BM smear (34).

Most patients in our series carried *UBA1* p.Met41 mutations, with p.Met41Thr being the most common, followed by Leu and Val variants, as previously reported (12, 21, 30). In addition, 2 mutations related to splicing were detected, and also the presence of the c.209T>A mutation (variant of uncertain significance), only described in Spain together with a clinical presentation consistent with VEXAS, standing out (31). *DNTM3A* and *TET2* were the most commonly co-mutated genes, as reported by Gutierrez-Rodrigues and colleagues (16). Previously described phenotype-genotype associations are partially reproduced in our cohort, such as the association between the Thr mutation and relapsing polychondritis (11) or eye involvement (4, 5). However, no further associations could be demonstrated, such as less fever and lung involvement in the Leu mutation or more MDS and R-CP in the Val mutation, previously described (6), which may be due to the small number of cases included. However, the clinical picture accompanying the c.209T>A uncertain significance mutation is reported although further studies on this mutation are still ongoing. Additionally, we report for the first time an association between age at diagnosis ≥70 years, lower prevalence of lymphadenopathy, and grade ≥2 anemia with the Leu mutation. Given the small number of patients in each mutation subgroup, these findings should be interpreted as exploratory, and the wide confidence intervals observed in some comparisons limit the robustness of the estimates. Therefore, these observations require validation in larger, independent cohorts.

This study illustrates the lack of standard treatment so far for the management of patients with VEXAS syndrome after a confirmed diagnosis, though different treatment algorithms have recently begun to be proposed (2, 18, 34).

Glucocorticoids are usually required to control inflammation in patients with VEXAS syndrome (18). In our cohort, 90% of patients received steroids, either alone or combined with other therapies, similar to the UK and French cohorts (21, 30). Given the frequent need for prolonged treatment, tapering must be performed slowly to reach the minimal effective dose while minimizing long-term toxicity (18). This approach is effective to initially control inflammation (23, 24) and in our cohort this effectiveness is reflected in the high rate of complete response observed in this subgroup of patients (57.1% at 6 months), also patients were steroids naïve in this setting. However, 47.1% of the initial patients had to discontinue glucocorticoid therapy, half due to inadequate disease control and the other half because of the need for steroid sparing. This finding underscores one of the major limitations of long-term glucocorticoid use in VEXAS: although they are highly effective for inducing an initial response, their long-term sustainability is poor due to cumulative toxicity and the difficulty in maintaining inflammatory control at reduced doses.

In our cohort, JAK inhibitors were initiated in patients with a more severe baseline profile, reflected by the highest inflammatory burden (C-RP 13.4 mg/dL, IQR, 7.8–26.0) and a greater frequency of transfusion dependence (4/11, 36.4%) compared with other treatment groups. These patients had also received more previous therapies (median 3 lines, range, 2–4), indicating later use in the therapeutic sequence. This clinical context likely contributed to the more modest responses observed, particularly when compared with the UK and French cohorts (21, 30), where JAK inhibitors were introduced earlier and in patients with lower inflammatory and hematologic burden. These differences in baseline severity and timing of therapy initiation provide a plausible explanation for the variability in outcomes and help contextualize the effectiveness of JAK inhibition in VEXAS syndrome.

Overall, responses to anti-IL1/IL6 therapy in our cohort were discrete, and given the very small number of treated patients and their heavily pretreated profile, no firm conclusions regarding their effectiveness can be drawn (21, 30).

We showed that HMA therapy was associated with relevant treatment responses, with any global response documented in 3 of 6 evaluable patients (50%) at 6 months and, at 12 months, complete responses in 3 of 6 patients (50%) and partial responses in 2 of 6 (33.3%). These patients had received a median of three previous treatment lines (range, 1–8), and most had an underlying myeloid neoplasm, predominantly MDS (6/12, 50%) and including one case of AML (1/12, 8.3%). Despite this heavily pretreated profile, treatment discontinuation was infrequent (2/11, 18.2%) and the median TNT-D reached 8 months (IQR, 2–13), supporting a favorable balance between clinical benefit and tolerability. These observations should be interpreted with caution because of the retrospective design, the relatively short follow-up, and the limited duration of azacitidine exposure—consistent with the early clinical experience accumulated following the recognition of VEXAS syndrome (25–27, 35, 36). Notably, the French phase II trial reported that first-line azacitidine combined with glucocorticoids achieved symptom control in all patients with concomitant MDS, with 66% clinical improvement, 60% hematologic responses, and successful steroid tapering below 10 mg/day (25). Similarly, a large retrospective cohort enriched for MDS (>80%) showed that azacitidine alone yielded inflammatory responses exceeding 50% at one year, relapse-free survival of 90%, transfusion independence in 65%, and a ≥25% reduction in VAF in more than half of patients (26). In line with the UK cohort (21), our findings support the potential role of HMA in VEXAS. These observations suggest a potential long-term benefit of HMA therapy in VEXAS, although the small number of treated patients precludes any comparative conclusions and should be interpreted as descriptive and findings require validation un larger, independent cohorts.

The prognosis of VEXAS syndrome disease remains poorly defined and it is considered a potentially life-threatening, with increased mortality (1, 15, 18, 32). The main reported cause of death reported in our series was severe infections (10%). Recently, the French VEXAS group reported a high incidence of serious infections in these patients, including atypical infections (37). The only factor associated with poorer survival was gastrointestinal involvement (HR = 8.753, 95% CI 1.444 - 53.044*, P* = 0.0.18), also described by the French group (5). Other biological and clinical characteristics previously suggested as prognostic (MDS, transfusion dependence, severe thrombocytopenia, or pMet41Val mutation) (1, 4, 5) could not be confirmed in our study, most likely due to the limited number of events and the small sample size. The only protective factor to predict OS identified in our cohort was response to treatment, both clinical and biological (*P* = 0.003 and *P* < 0.001, respectively).

The duration of treatment in our cohort showed meaningful variation across therapeutic classes, reflecting differences in how these agents are used throughout the disease course. The extended TNT-D with corticosteroids in monotherapy (median 9 months) likely reflects their capacity to induce a rapid and reliable initial response, particularly in patients with prominent inflammatory manifestations. In contrast, the prolonged TNT-D observed with HMA (median 8 months) appears to reflect a more sustained effect, consistent with their potential to modulate the underlying clonal process (18). No clinical, analytical, or genetic predictors of treatment discontinuation were identified. Importantly, the small sample size limits the strength of these observations, and no definitive conclusions can be drawn. Larger cohorts and longer follow-up will be essential to clarify whether these patterns reflect true differences in treatment durability or simply variability inherent to early real-world experience.

The present study has several limitations, particularly the sample size, the retrospective nature and the short follow-up after genetic diagnosis. In addition, most of the patients come from hematology consultations, so the sample is enriched with MDS patients. Also, many patients received different treatments prior to genetic diagnosis, especially corticosteroids, and it is difficult to assess their impact on the different endpoints. The sub-analyses performed included few patients, so interpretations should be made with caution. However, this multicenter study provides novel scientific evidence on a new disease for which there are limited clinical data, including the outcomes of these patients after different therapeutic approaches, and illustrating the variability in management strategies and the use of multiple treatment approaches during the initial years following the discovery of VEXAS syndrome.

In summary, the clinical and biological features of our cohort are consistent with those previously reported. We described clinical characteristics of the c.209T>A mutation (variant of uncertain significance), as well as a new clinical phenotype of patients with the Leu mutation, who show less lymphadenopathy and lower degree of anemia and are diagnosed older. Treatment patterns in our cohort illustrate how different therapeutic classes are used across the disease course, with steroid-monotherapy regimens frequently providing early symptom control and HMA may also be useful. The response to treatment and the presence of gastrointestinal involvement emerged as potential prognostic signals, although these observations should be interpreted cautiously. Patterns of treatment use and the observed TNT-D provide a descriptive overview of how different therapeutic approaches perform across the disease course. Given the small sample size and the descriptive nature of our analyses, no conclusions regarding comparative effectiveness can be drawn. These findings require validation in larger external cohorts with longer follow-up.

## Data Availability

The raw data supporting the conclusions of this article will be made available by the authors, without undue reservation.

## Group member of Grupo Español de Síndromes Mielodisplasicos (GESMD)

Alba Mesa-Tudela, Ana Villalba, Borja Puertas, Carlos De Miguel, Carlos Puerta, Daniel Gil-Alós, Dolly Fiallo-Suarez, Esperanza Tuset, Felix López-Cadenas, Fernando Ramos, Idoya Ancin-Arteaga, Javier Arzuaga-Méndez, Jesus María Hernandez-Rivas, Karan Mayani, M. Ángeles Montañes-García, Marc Bosch-Schips, María Díez-Campelo, María Laura Blanco, María Moreno-Igoa, Maria Teresa Gimenez, Marina Diaz-Beya, Marta Canet-Maldonado, Marta Fonseca-Santos, Pablo Palomo-Rumschisky, Raúl Azibeiro, Sandra Castaño Diez, Valeria Peri.
