# Supplementary material for: Clinical, laboratory and genetic characteristics of VEXAS syndrome: a study on behalf of GESMD
Source: Front Immunol. 2026 Apr 29;17:1743509. doi: 10.3389/fimmu.2026.1743509 (PMC13168092; doi:10.3389/fimmu.2026.1743509)
Supplement: Supplementary file 1 [file Table1.docx]

**SUPPLEMENTARY MATERIAL**

Table S1. Clinical onset of VEXAS syndrome.

| **Clinical onset** | **All patients** **(N=40)** |
| --- | --- |
| **Cutaneous, n (%)**  Neutrophilic dermatitis, n  Not specified, n  Erythema nodosum, n | 17 (42.5)  11  5  1 |
| **Rheumatologic, n (%)**  Auricular and/or nasal chondritis, n  Polymyalgia rheumatica, n  Polyarthritis, n  Polyarteritis nodosa,  Seronegative rheumatoid arthritis, n  Migratory polyarthralgia, n | 12 (30.0)  5  3  1  1  1  1 |
| **General syndrome, n (%)**  Fever of unknown origin, n  Asthenia and weight loss, n | 5 (12.5)  4  1 |
| **Hematologic alterations, n (%)**  Anemia, n | 3 (7.5)  3 |
| **Thrombosis, n (%)**  Deep venous thrombosis, n  Pulmonary thromboembolism, n | 2 (5.0)  1  1 |
| **Pulmonary, n (%)**  Organizing pneumonia, n | 1 (2.5)  1 |

Table S2. Mutations detected by Next Generation Sequencing in patients with VEXAS syndrome.

| **Mutations*** | **n (%)** |
| --- | --- |
| *UBA1* alone | 20 (66.7) |
| *TET2* | 4 (13.3) |
| *DNMT3A* | 3 (10.0) |
| *PTRF8* | 1 (3.3) |
| *SH2B3* | 1 (3.3) |
| *MPL* | 1 (3.3) |
| *ASXL1* | 1 (3.3) |
| *UA2F1* | 1 (3.3) |
| *RUNX1* | 1 (3.3) |
| *ANKRD26* | 1 (3.3) |

**Next generation sequencing was carried out in 30 of 40 patients.*

Table S3. Lines of treatment used after genetic confirmation of VEXAS

| **All patients (N=17)**  **Overall treatment lines= 58** | | | |
| --- | --- | --- | --- |
| Therapeutic cathegory, N (%) | Treatment | Line of therapy | N (%) |
| Steroids-based therapy, 17 (31.0) | Prednisone/Prednisolone | First | 14 (24.3) |
|  | Methylprednisolone bolus | First | 3 (5.2) |
| Jak inhibitor-based therapy  N=11 | Upadacitinib plus prednisolone | First | 1 (1.8) |
|  | Ruxolitinib plus prednisolone | First | 6 (10.4) |
|  |  | Second | 3 (5.3) |
|  | Baricitinib plus prednisolone | Second | 1 (1.8) |
| Anti-IL1 and 6 based therapy  N= 7 (12.1%) | Anakinra alone | First | 1 (1.8) |
|  | Anakinra plus prednisolone | Second | 3 (5.3) |
|  | Canakinumab plus prednisolone | First | 1 (1.8) |
|  | Tocilizumab plus prednisolone | Second | 2 (3.5) |
| Hypomethylating-based  therapy N=12 | Azacitidine alone | First | 1 (1.8) |
|  |  | Third | 1 (1.8) |
|  | Azacitidine plus prednisolone | First | 4 (7.0) |
|  |  | Second | 2 (3.5) |
|  |  | Third | 2 (3.5) |
|  | Azacitidine plus Venetoclax | First | 1 (1.8) |
|  | Decitabine plus prednisolone | Second | 1(1.8) |
| Others N=10 | Methotrexate plus prednisolone | First | 4 (7.0) |
|  |  | Second | 2 (3.5) |
|  | Hydroxychloroquine plus prednisolone | First | 2 (3.5) |
|  |  | Second | 1 (1.8) |
|  | Cyclosporine | Second | 1 (1.8) |

Figure S1. Overall survival


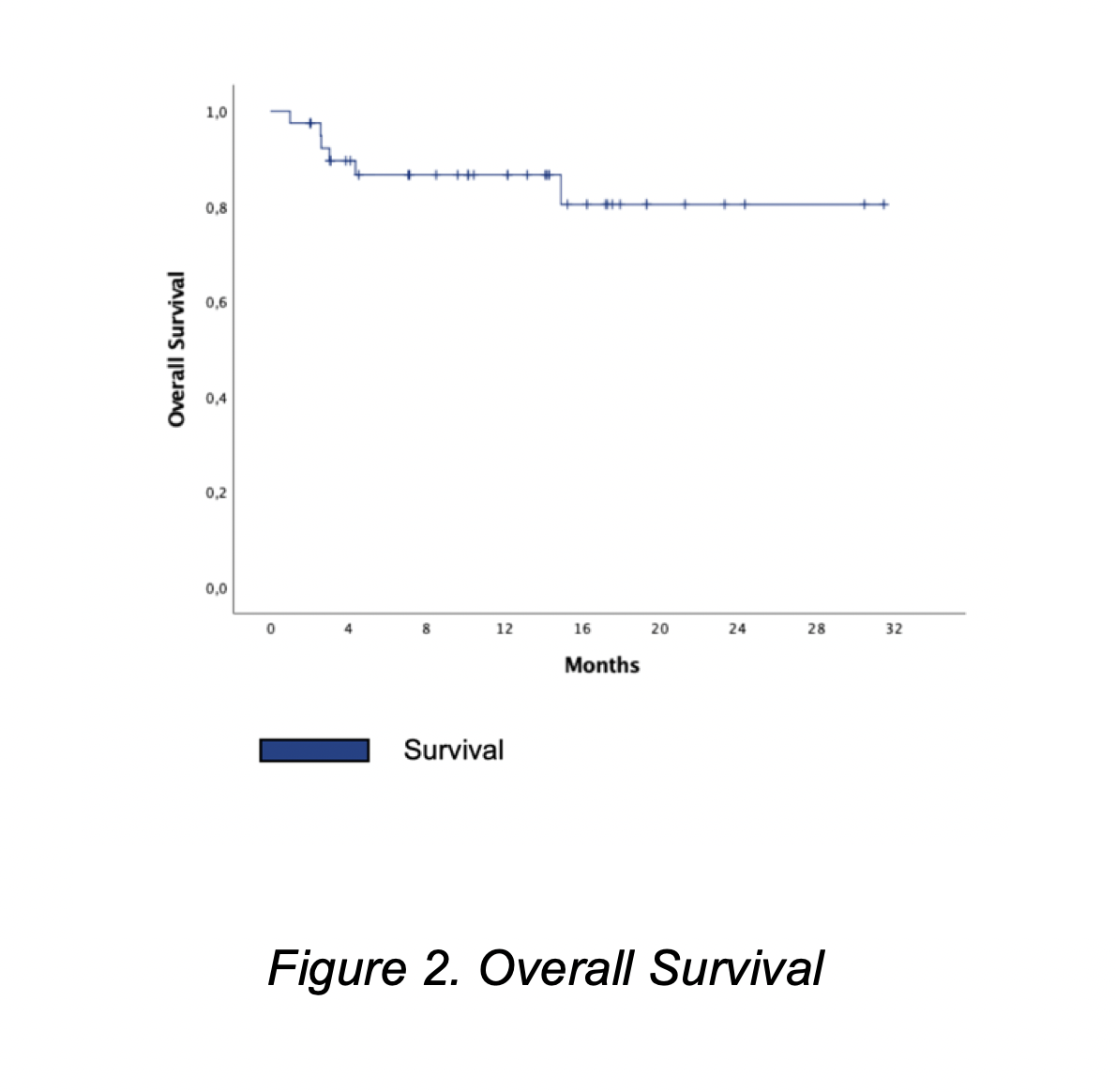
.

Table S4. Risk analysis for OS

| Risk factors | Univariate analysis | | |
| --- | --- | --- | --- |
|  | *p* | HR | CI 95% |
| Age ≥ 70 years | 0.699 | 0.730 | 0.147-3.616 |
| pMet41Val | 0.506 | 0.038 | 0.000-577 |
| p.Met41Thr | 0.815 | 1.212 | 0.242-6.069 |
| p.Met41Leu | 0.630 | 0.589 | 0.068-5.090 |
| Organs affected ≥4 | 0.928 | 1.082 | 0.196-5.977 |
| Diagnosis < 1 year of onset symptoms | 0.549 | 1.672 | 0.306-9.134 |
| Skin involvement | 0.997 | 0.996 | 0.115-8.655 |
| Lung involvement | 0.316 | 3.009 | 0.350-25.881 |
| Chondral involvement | 0.841 | 0.845 | 0.163-4.381 |
| Arthritis | 0.645 | 0.685 | 0.137-3.419 |
| Thrombosis | 0.441 | 1.881 | 0.377-9.380 |
| Cardiac involvement | 0.678 | 1.576 | 0.184-13.531 |
| Arterial involvement | 0.676 | 0.045 | 0.000-Inf |
| Kidney involvement | 0.202 | 4.167 | 0.465-37-348 |
| Eye involvement | 0.601 | 0.633 | 0.114-3.508 |
| Polyneuropathy | 0.631 | 0.044 | 0.000-Inf |
| Gastrointestinal involvement | **0.018** | **8.753** | **1.444-53.044** |
| Testicular involvement | 0.656 | 0.043 | 0.000-Inf |
| Adenopathy | 0.695 | 1.412 | 0.252-7.909 |
| C-RP≥10 mg/dL | 0.153 | 3.454 | 0.630-18.931 |
| ESR ≥ 50 | 0.486 | 31.830 | 0.002-Inf |
| Ferritin ≥ 1500 | 0.096 | 6.228 | 0.725-53.526 |
| Grade ≥ 2 Anemia | 0.397 | 2.537 | 0.294-21.893 |
| Transfusion dependency | 0.295 | 2.354 | 0.474-11.703 |
| Grade ≥ 2 Neutropenia | 0.076 | 7.332 | 0.810-66.353 |
| Grade ≥ 2 Lymphopenia | 0.793 | 1.257 | 0.229-6.901 |
| Grade ≥ 2 Thrombocytopenia | 0.859 | 0.823 | 0.096-7.050 |
| MDS | 0.178 | 0.229 | 0.027-1.959 |
| MGUS | 0.735 | 0.046 | 0.000-Inf |
| MBL | 0.800 | 0.048 | 0.000-Inf |
| HL | 0.800 | 0.048 | 0.000-Inf |
| AML | 1.000 | 1.000 | 0.000-Inf |
| Lines of treatment ≥ 2 | 0.742 | 1.311 | 0.261-6.584 |
| CR to treatment | **0.001** | **NC*** | **NC*** |
| BR to treatment | **0.003** | **NC*** | **NC*** |

*NC: not calculable

| Risk factors | Univariate analysis | | |
| --- | --- | --- | --- |
|  | *p* | HR | CI 95% |
| Age ≥ 70 years | 0.312 | 0.659 | 0.294-1.148 |
| pMet41Val | 0.711 | 0.795 | 0.237-2.670 |
| p.Met41Thr | 0.968 | 1.106 | 0.463-2.230 |
| p.Met41Leu | 0.442 | 0.655 | 0.223-1.925 |
| Organs affected ≥4 | 0.927 | 0.960 | 0.400-2.303 |
| Diagnosis < 1 year of onset symptoms | 0.378 | 0.643 | 0.241-1.716 |
| Skin involvement | 0.982 | 0.986 | 0.293-3.317 |
| Lung involvement | 0.755 | 1.027 | 0.868-1-216 |
| Chondral involvement | 0.479 | 1.343 | 0.593-3.041 |
| Arthritis | 0.447 | 1.376 | 0.604-3.136 |
| Thrombosis | 0.554 | 1.275 | 0.570-2.851 |
| Cardiac involvement | 0.720 | 0.767 | 0.180-3.274 |
| Arterial involvement | 0.811 | 1.194 | 0.279-5.100 |
| Kidney involvement | 0.464 | 1.727 | 0.401-7.447 |
| Eye involvement | 0.858 | 0.931 | 0.423-2.047 |
| Polyneuropathy | 0.395 | 0.418 | 0.056-3.115 |
| Gastrointestinal involvement | 0.174 | 2.119 | 0.718-6.523 |
| Testicular involvement | 0.474 | 0.481 | 0.065-3.579 |
| Adenopathy | 0.355 | 0.604 | 0.207-1.761 |
| C-RP≥10 mg/dL | 0.280 | 1.549 | 0.701-3.424 |
| ESR ≥ 50 | 0.829 | 0.906 | 0.372-2.208 |
| Ferritin ≥ 1500 | 0.374 | 0.613 | 0.208-1.804 |
| Grade ≥ 2 Anemia | 0.940 | 0.968 | 0.410-2.282 |
| Transfusion dependency | 0.344 | 1.511 | 0.643-3.552 |
| Grade ≥ 2 Neutropenia | 0.537 | 0.047 | 0.000-788 |
| Grade ≥ 2 Lymphopenia | 0.394 | 0.628 | 0.215-1.830 |
| Grade ≥ 2 Thrombocytopenia | 0.218 | 0.504 | 0.170-1.498 |
| MDS | 0.125 | 1.847 | 0.027-1.959 |
| MGUS | 0.176 | 2.749 | 0.635-11.903 |
| MBL | 0.583 | 0.047 | 0.000-251 |
| HL | 0.506 | 0.044 | 0.000-433 |
| AML | 0.721 | 0.048 | 0.000-841 |
| Lines of treatment ≥ 2 | 0.415 | 1.465 | 0.596-3.598 |
| Steroid based therapy | 0.580 | 0.787 | 0.337-1.840 |
| JAK inhibitors based therapy | 0.419 | 1.465 | 0.580-3.702 |
| Hypomethilant based therapy | 0.628 | 0.766 | 0.261-2.248 |
| Anti-Il1 and IL6 based therapy | 0.873 | 0.888 | 0.209-3.783 |
| Complete response at 6 months | 0.469 | 0.030 | 000-386 |
| Any response at 6 months | 0.782 | 1.377 | 0.143-113.287 |

Table S5. Risk analysis for TNT-D survival
